# Supplementary material for: Rasch-Validated Italian Scale for Diagnosing Digital Eye Strain: The Computer Vision Syndrome Questionnaire IT©
Source: Int J Environ Res Public Health. 2022 Apr 8;19(8):4506. doi: 10.3390/ijerph19084506 (PMC9028942; doi:10.3390/ijerph19084506)
Supplement: Supplementary file 1 [file ijerph-19-04506-s001.zip › ijerph-1603111-supplementary.pdf]

CVS-Q© IT

(COMPUTER VISION SYNDROME QUESTIONNAIRE)

QUESTIONARIO PER LO STUDIO DELLA SINDROME DA VISIONE AL COMPUTER

Da compilare a cura del lavoratore

Indichi se percepisce qualcuno dei seguenti sintomi, durante l'utilizzo del computer nel lavoro.
Per ogni sintomo, segni con una X:

a. In primo luogo, la frequenza con la quale appare il sintomo, tenendo conto che:
   - MAI = in nessuna occasione
   - OCCASIONALMENTE = in forma sporadica o una volta alla settimana
   - DI SOLITO O SEMPRE = 2 o 3 volte alla settimana o quasi tutti i giorni

b. In secondo luogo, l'intensità con la quale sente il sintomo:
   - Ricordi: se segnala "MAI" in frequenza, non deve compilare l'intensità.

	**a. Frequenza**			**b. Intensità**	
	MAI	OCCASIONALMENTE	DI SOLITO O SEMPRE	MODERATA	INTENSA
1. Bruciore					
2. Pizzicore					
3. Sensazione di corpo estraneo					
4. Lacrimazione					
5. Ammiccamento eccessivo					
6. Arrossamento oculare					
7. Dolore oculare					
8. Pesantezza delle palpebre					
9. Secchezza					
10. Visione sfuocata					
11. Visione doppia					
12. Difficoltà nella messa a fuoco da vicino					
13. Aumento di sensibilitá alla luce					
14. Aloni di colori intorno agli oggetti					
15. Sensazione di vedere peggio					
16. Mal di testa					

Da compilare a cura del ricercatore

Calcolo del PUNTEGGIO TOTALE considerando che:

- Frequenza:
  - MAI = 0
  - OCCASIONALMENTE =1
  - DI SOLITO O SEMPRE = 2
- Intensità:
  - MODERATA =1
  - INTENSA = 2
- Gravità:
  - Il risultato di frequenza per intensità deve essere ricodificato come: 0 = 0; 1 o 2 = 1; 4 = 2

	Frequenza	Intensità	Frequenza x Intensità	Gravità
1. Bruciore				
2. Pizzicore				
3. Sensazione di corpo estraneo				
4. Lacrimazione				
5. Ammiccamento eccessivo				
6. Arrossamento oculare				
7. Dolore oculare				
8. Pesantezza delle palpebre				
9. Secchezza				
10. Visione sfuocata				
11. Visione doppia				
12. Difficoltà nella messa a fuoco da vicino				
13. Aumento di sensibilità alla luce				
14. Aloni di colori intorno agli oggetti				
15. Sensazione di vedere peggio				
16. Mal di testa				

$$\text{Punteggio totale} = \sum_{i=1}^{16}$$

Se il punteggio totale è ≥ 7 punti, il lavoratore è affetto da Sindrome da Visione al Compute (Computer Vision Syndrome).
